# Evaluation of Chronic Disease Prevention and Control Public Service Advertisement on the Awareness and Attitude Change among Urban Population in Chongqing, China: A Cross-Sectional Study

**DOI:** 10.3390/ijerph14121515

**Published:** 2017-12-05

**Authors:** Tingting Wu, Ping Hu, Hao Huang, Chengbin Wu, Zhirong Fu, Lei Du, Xianglong Xu, Zumin Shi, Yong Zhao

**Affiliations:** 1 School of Public Health and Management, Chongqing Medical University, Chongqing 400016, China; m17749945967@163.com (T.W.); m18323165365@163.com (P.H.); xianglong1989@126.com (X.X.); 2 Research Center for Medicine and Social Development, Chongqing Medical University, Chongqing 400016, China; 3 Collaborative Innovation Center of Social Risks Governance in Health, Chongqing Medical University, Chongqing 400016, China; 4 Chengdu Blood Center, Chengdu 610000, China; 5 Institute of Health Education, Chongqing 401120, China; hhcqhei9709@163.com (H.H.); need591741@163.com (C.W.); fuzhirong_1019@163.com (Z.F.); 13983269778@163.com (L.D.); 6 Adelaide Medical School, University of Adelaide, Adelaide 5000, Australia; zumin.shi@adelaide.edu.au

**Keywords:** public service advertisement, chronic diseases, awareness

## Abstract

The aim of this study is to evaluate the influence of public service advertising on the awareness and attitude of Chongqing urban citizens. The theme of the public service advertisement launched in Chongqing was chronic disease prevention and control. A self-designed questionnaire was used in an outdoor intercept survey to collect information about the perception of citizens toward the effect of the advertisement on awareness and attitude situation. Respondents had good knowledge of chronic disease (17.11 ± 3.23, total score: 23), but only 58.4% of participants thought cancer is one type of chronic disease. The awareness of cancer as a chronic disease among the group who had seen this advertisement (63.6%) was higher than that of the group who had not seen the advertisement (56.5%) (*p* = 0.046). The attitude of respondents was good after watching the advertisement, approximately 77.4% of respondents attempted to remind their family and friends to prevent chronic diseases, roughly. 78.2% tried to persuade their family and friends to change their unhealthy lifestyle habits, and 84.7% of participants reported that the advertising increased the possibility of their own future lifestyle change. There was minimal change of awareness of the participants who saw the advertisement. This study did not show significant differences on chronic disease related knowledge between the participants who have seen the advertisement and who have not seen the advertisement. The public service advertisement may help participants improve the attitude of future behavior change. Further researches combining the sustained intervention and support through clinical and community health programs media campaigns are needed to support public health.

## 1. Introduction

With the developing social economy and increasing aging population, the incidences of chronic diseases (e.g., cardiovascular and cerebrovascular diseases, diabetes, and chronic obstructive pulmonary disease (COPD)) are dramatically increasing, which pose a serious threat to people’s lives and health [1]. The effects of chronic diseases on quality of life are also inevitable [2]. Chronic diseases have become a global public health problem [3]. According to the statistics released by China’s Ministry of Health, chronic diseases affect more than 260 million people in China, which accounts for 85% of deaths per year and 70% of the disease burdens [4]. Chronic diseases in China will enter a “blowout phase” in the next 30 years [5,6].

Chronic diseases have already posed a substantial economic burden. In 2005, the World Health Organization (WHO) projected that Asia would experience the largest increase in death rates from cardiovascular disease, cancer, respiratory disease, and diabetes over the next 10 years. China, India, and the Russian Federation could lose billions of dollars in national income over the next 10 years [7]. In China, the estimated accumulated losses from 2005 to 2015 amount to US $558 billion [8]. In 2011, the World Bank estimated that without effective measures, the disease burden caused by myocardial infarction, stroke, diabetes, and COPD would increase to more than 50% of all disease burdens in the next two decades [9]. In particular, the chronic disease burden in China would increase by 40% because of the rapid aging population [10].

Faced with the growing serious problem of chronic disease prevention, and the unoptimistic view of the chronic disease situation [11,12,13,14], taking measures to control the incidences of chronic disease in China is crucial. Authorities can carry out additional measures to improve knowledge and change attitudes and behaviors of the citizens toward chronic disease prevention. The unhealthy lifestyle and factors are the main events influencing of chronic diseases [15]. The effective health education and health promotion, to a certain extent, can change individuals’ unhealthy lifestyles and ultimately reduce the incidences of chronic diseases [16].

Mass media interventions often involve campaigns by television, radio, newspapers, billboards, posters, leaflets, or booklets. Public interest advertising, which acts as a high-level advertising form and an independent social education power, is an important approach to health education and promotion, which is called “advertising conscience” [17,18]. Public service advertisements are usually used to alert the public of important health issues [19,20,21,22]. Behavior change following public service advertising has been observed in many health campaigns, such as those on melanoma prevention programs [23], gynecologic cancer [24], and physical activity [25,26,27,28]. Mostly, a conscious effort to stop smoking, which is a preventable risk factor for people with chronic diseases, is observed [29,30]. These campaigns have achieved good results, and public interest advertising is considered an effective means to change the public’s awareness and behavior.

From 16 November 2012 to 31 December 2013, the public service advertisement “being healthy, being away from chronic diseases” produced by the Health Education Institute in Chongqing was broadcasted through television (it means the general television at home, like Cable TV) and mobile television (including the TV on the bus, the ferry, the rail transit). The advertisement entitled “being healthy, being away from chronic diseases” intended to raise public awareness and motivate public to change unhealthy behavior. This study aimed to evaluate the influence of this public service advertising on the awareness of Chongqing citizens, and to assess perceived impact of the knowledge and likely future behaviors of people.

## 2. Materials and Methods

### 2.1. Method and Participants

We selected 250 people each from four communities among the main city zones of Chongqing to participate in an outdoor intercept survey. To meet a diverse set of people, we chose the places where two to five people often socialize within the community, such as the city square, department stores, railway stations, hospitals, schools, and markets.

Participants who met the following inclusion criteria were included: (1) literate, (2) aged 24–60, and (3) Chongqing local who lived there for at least half a year.

### 2.2. Ethics Committees

This project was reviewed and approved by the Ethical Committee of the Chongqing Medical University and Chongqing Health Education Institute (No. 2016001). Oral consent was obtained from all participants. The participants were also informed that they could withdraw from the study at any stage.

### 2.3. Public Service Advertisement

The public service advertisement entitled “being healthy, being away from chronic diseases” includes three sections: (1) common types of chronic diseases (e.g., cardiovascular and cerebrovascular diseases, diabetes, and chronic obstructive pulmonary disease); (2) important influencing factors (hyperlipidemia, hypertension, unhealthy diet, smoking, excessive drinking, lack of physical exercise, overweight, obesity, etc.); (3) and some preventive measures (e.g., good nutrition, physical activity, quieting smoking and controlling drinking, healthy eating, healthy state of mind). It is a 30 s long animated video with audio.

This advertisement was broadcasted by the television station as well as mobile bus or subway in Chongqing according to the following schedule: (1) from 16 November 2012 to 30 July 2013, the advertisement was aired on “Chongqing news” at 6:30 a.m. every day and then replayed on the news channel three times: on the same day at 10:00 p.m. and on the next day at 1:29 and 3:36 a.m., which totals to 1020 times (4 × 30 × 8.5 = 1020). (2) From 1 March to 30 June 2013, Chongqing mobile television channel broadcasted the advertisement twice in the morning, at noon, and in the afternoon, every day, which amounts to 540 times (6 × 30 × 3 = 540). (3) From 1 June to 31 December 2013, the advertisement was broadcasted 2–3 times during “Healthy Hour” in the morning every day. From Monday to Friday, it was also broadcasted once before “Bu Jian Bu San” at 7:30 p.m., which equals 630 times (3 × 30 × 7 = 630). In total, the advertisement was broadcasted 2190 times (1020 + 540 + 630 = 2190) during the entire period. The “Chongqing news” is a program of newscast and the “Healthy Hour” is a health promotion program on Chongqing TV. The two programs were both broadcasted on television and mobile television. There was no other public service advertisement broadcasted in Chongqing during the same period.

### 2.4. Questionnaire

Our survey used a self-designed questionnaire based on a literature review of related local and international studies [31,32,33,34,35]. The final questionnaire was obtained after repeated discussions with experts and a pilot study, which had an acceptable level of face and content validity and readability. The pilot test was conducted in a medical university. A total of 30 individuals participated in this pretest. We modified the questionnaire according to results of the pilot test, especially on the presentation of questions.

All the questions of questionnaires are close-ended. It had three sections: (1) Demographic characteristics; (2) Knowledge about types of Chronic disease, the influence factor of chronic disease and healthy lifestyle of prevent chronic diseases; (3) Effect measure questions of the chronic disease prevention and control advertising. Recognizing the advertisement was defined based on the “yes” answer of respondents to “Have you seen the chronic disease prevention and control advertising?”, while the investigators was giving the video information of the advertising. Before the participants answered whether they had saw the advertisement, they were required to answer the daily media exposure frequency.

Demographic characteristics included name, age (categorized as 25–44 years and 44–50 years), educational level, occupation, residence time (categorized as six months to one year and above one year) were assessed by self-reporting. Education levels were categorized as primary school or below, junior middle school (basic education), senior high school (including vocational/technical secondary school and junior college, and secondary education) and senior college and university (higher education) or above. Occupation was categorized as students, famers, labors, office staff (including government employee and private employee) and professional job (including teachers and medical staff).

For the knowledge section, 23 questions were included about the types of chronic disease, causes of chronic disease, healthy lifestyle for chronic disease prevention, concepts of chronic disease prevention, knowledge of hypertension and diabetes prevention, harm of smoking or secondhand smoking. A question answered correctly scored 1 point. Incorrect, unsure, or missing responses scored 0. The maximum score from the 23 knowledge questions was 23 units. The total score of knowledge was classified into three categories on the basis of the quintile scores and coded as “low”, “good”, and “excellent” for ≤60%, 61% to 80%, and >80%, respectively.

Regarding with the effect measure of the chronic disease prevention and control advertising, the participants who had seen the advertising were required to answer the following questions to reflect the personal subjective assessment of the immediate and follow-ups effects of the public service advertising: (1) Has the advertising made you stop and think? (Yes/No); (2) Was the advertising relevant to your life and you? (Yes/No); (3) Has the advertising provided new information to you? (Yes/No); (4) Have you said something about the advertising or discussed it with others after you watching the advertising? (Yes/No); (5) Has the advertising made you pay more attention to the risk factors for chronic diseases? (Yes/No); (6) Have you tried to remind others to prevent chronic disease after watching the advertising? (Yes/No); (7) Have you tried to persuade others to change their unhealthy lifestyle after watching the advertising? (Yes/No); (8) Has the advertising made you more likely to take step to change your lifestyle? (Yes/No).

### 2.5. Investigation Method

Data collection was conducted about three days after the end of the advertising broadcasted. At each data collection point, each team needed at least two investigators to get the most type and number of people, but each interview was conducted one on one. The timing of data acquisition was arranged during different times of the day, in the mornings, afternoons, or late evenings (twice a day). For a unified format of inviting people to attend the interview, the interviewer was required to say “hello” and invite every fifth passerby to participate in the survey. After a self-introduction, the interviewer showed the certificate of survey and explained the purpose of the survey. Finally, anonymous questionnaires were used to collect information with the respondents’ informed consent.

Regarding the questionnaire, each was checked by the members of the team on the basis of whether all answers were consistent, complete and true. Then, the team members gave a uniform number to the valid questionnaires.

### 2.6. Evaluation Method

Nearly 1 in 3 (27%) of the respondents reported that they have seen the advertisement. The advertising message was appraised as intended, as well as the attitude about the future behavior change. The vast majority of participants said that the advertisement was easy to understand, personally relevant, provided new information, and made them stop and think. When participants are willing to discuss this knowledge with others, or to persuade others pay attention to the harm of chronic, which may prove that they are willing to change their behavior, and even they have changed their behavior.

### 2.7. Quality Control

The survey was chaired by the Chongqing Health Education Institute, which is responsible for developing a unified survey program and preparing training materials for the site survey work to provide technical support and advice. Investigators underwent unified training and conducted pre-investigation. During the actual investigation, the Chongqing Health Education Institute organized personnel to conduct fieldwork supervision and quality control. Strict quality control measures were implemented to ensure that the survey data reflects the true situation as objectively as possible. Quality control was conducted throughout the entire investigation, including program design, training, site surveys, data management, and analysis.

### 2.8. Statistical Analysis

Data from the questionnaires were double checked carefully before entry into the database using Epidata 3.02 software (EpiData Association, Odense, Denmark). After strict sorting, data cleaning and analyses were conducted using statistical analysis system software (version 9.2; SAS Institute, Cary, NC, USA). Descriptive data were expressed as mean and standard deviation (SD) or percentage (%). Analysis of *t*-test was used to ascertain the significance of differences between continuous variables. Chi-square test was used to test differences of categorical variables between two groups. Logistic regression was used to assess the association between factors and behavior change after watching the advertisement. All statistical tests were performed using a two-sided test, and a *p*-value ≤ 0.05 was considered statistically significant.

## 3. Results

### 3.1. Demographic Characteristics of Participants

A total of 1089 individuals were approached for this survey, but 89 did not meet the requirements (42 and 29 did not meet the requirements for age and place of residence, respectively. An additional 18 withdrew from the study). A total of 1000 questionnaires were issued to qualified people, but there were 15 participants did not finish all the question, which were regarded as the invalid questionnaires. The total of 985 valid questionnaires were received.

The demographic characteristics of participants are provided in Table 1. All participants were aged between 24 and 60 years (24–44: 57.7%, 45–60: 42.3%) and lived in Chongqing for at least half a year (6–12 months: 8.8%, more than 12 months: 91.2%). All participants were divided into two groups (group 1: had seen the advertisement, group 2: had not seen the advertisement); only 261 (26.5%) reported that they have seen the advertisement. No significant difference was noted between the demographic characteristics of the two groups.

### 3.2. Chronic Disease Knowledge

The score of chronic diseases knowledge gained by participants who have seen the advertisement is “excellent”, while the score of the participants who have not seen the advertisement is “good” (Table 2). No significant difference existed between the two groups (18.02 ± 3.12 vs. 17.60 ± 3.26, *p* > 0.05).

The participants had a high awareness accuracy on certain chronic disease knowledge, and no significant difference was observed between the two groups: (1) cause of chronic disease (Hyperlipidemia, hypertension, hyperglycemia: 84.9%; Overweight and obesity: 83.4%; Smoking: 86.7%; Unhealthy diet: 89.6%; Lack of exercise: 87.7%; Excessive Drinking: 88.8%. *p* > 0.05); (2) correct concept of chronic disease prevention (89.8% of participants chose the correct concept, that is, “the sooner the better, throughout his life”, *p* > 0.05); (3) measures of hypertension prevention (87.7~93.7%, *p* > 0.05); and (4) measures of diabetes prevention (85.1~89.9%, *p* > 0.05). 

The participants had high awareness accuracy on most types of chronic disease (Cardiovascular disease: 71.4%; Diabetes: 80.0%; Chronic respiratory disease: 83.8%), and no significant difference existed between the two groups (*p* > 0.05). The participants’ awareness accuracy of cancer is low (Cancer: 58.4%), and the awareness of the group who had seen the advertisement (63.6%) was higher than that of the group who had not seen the advertisement (56.5%; *p* = 0.046). In addition, the participants also had high awareness accuracy on the lifestyle of chronic disease prevention (Quit smoking-limited wine: 92.5%; Balanced diet: 92.2%; Regular schedule: 92.9%; Regular exercise: 94.0%; Healthy state of mind: 86.3%), and no significant difference was noted between the two groups (*p* > 0.05). Only 35.33% of participants thought that healthy eating was a good lifestyle choice to prevent chronic disease, and awareness about healthy eating in the group who had seen the advertisement (47.1%) was higher than that of the group who had not seen the advertisement (31.1%; *p <* 0.001) (Table 3).

### 3.3. Personal Subjective Assessment of Effects of the Public Service Advertising

A total of 69.0%, 72.8%, and 74.0% of participants reported that “advertising is related to my life”, “advertising makes me stop and think about my life”, and “advertising provides new information to me”, respectively. After watching the advertisement, 63.2% of participants discussed it with others, 78.2% noticed the risk factors of chronic diseases, 77.4% tried to remind others to prevent chronic diseases, 78.2% tried to persuade others to change their unhealthy lifestyle, and 84.7% mentioned that it increased the possibility of changing their own unhealthy lifestyle (Table 4).

After its broadcast, the advertising caused some changes to participants, such as: (1) Discussed with others about this advertising; (2) Tried to remind others to prevent chronic diseases; and (3) Tried to persuade others to change their unhealthy style; (4) More likely to change healthy lifestyle. Several factors were considered in the modeling of the effects of the “being healthy, being away from chronic diseases” advertising, including gender, age, residential time, having chronic diseases, occupation, education level.

Table 5 shows the logistic regression analysis of the public service advertisement toward the advertisement effects, which indicates the participants whose job are professional more likely to discuss the advertising with others than students, famer, labor, and office staff (odds ratio (OR) = 2.412, 95% confidence interval (CI; 1.090–5.338); *p* = 0.030). And after watching the advertising, the office staff and farmer were more likely to change their future unhealthy lifestyle (OR = 0.293, 95% CI (0.105–0.822), *p* = 0.020; OR = 0.078, 95% CI (0.007–0.884), *p* = 0.039). But there was no significant difference between the four changes and the age, gender, and residence time of the respondents and whether have chronic disease.

### 3.4. Daily Contact of Mass Media of All Paticipants

According to the survey, the daily contact of mass media of the population is 59.8%, 42.5%, and 43.2% for television, website and social media, respectively. There was no significant difference in daily contact of these three medias existed between the two groups (*p* > 0.05), but for newspapers and magazine, significant difference was present between the two groups (*p* = 0.010 and 0.002) (Table 6).

## 4. Discussion

According to this survey, the participants have high awareness accuracy on the knowledge on chronic disease, including the types and causes of chronic disease, the healthy lifestyle and concepts of chronic disease prevention, the knowledge of hypertension and diabetes prevention, and the harm of smoking or secondhand smoking. There was no significant difference existed between the participants who have seen or have not seen the advertising. This result conforms to the finding of a study carried out in the Banan District of Chongqing [14]. Such outcome may be related to the previous implementations of the management and prevention of chronic diseases in Chongqing [36]. And in 2010, Chongqing began to establish the demonstration pilots for chronic diseases, and the comprehensive control and prevention of chronic diseases in the demonstration pilots were effective, and the remarkable improvement on the levels of health knowledge [37].

Only over half of the participants recognize cancer as a chronic disease in this study, the awareness rate of cancer is low, which is similar with the study in Xiaogan, China [38]. One-third thought healthy eating was a good lifestyle choice to prevent chronic disease. The two items rate of the group who had seen the advertisement was higher than that of the group who had not seen it. This may indicate the public service advertising have a minimal change for the participants awareness on these two items. Continued health education is necessary in Chongqing, especially the knowledge where the citizen easily confused and neglected.

The rates of chronic diseases in this study were answered by face-to-face interviews, more than 70% of participants reported that they don’t have a chronic disease. In China, people rarely take the initiative to have medical checkups until they feel unwell [39,40]. Which may lead them to a poor access to health care, screening and diagnosis of chronic disease. Most people may not know whether or not they actually suffer from chronic diseases due to no symptoms of obvious pain which might lead to a lower prevalence of chronic disease reporting. Further researches need to combine with clinical and community health programs to screen, treat and support positive behavior changes for chronic disease prevention.

Only 26.5% of respondents have seen the advertisement “being healthy, being away from chronic diseases”. However, the result did not fail the expectation, because considering the large population in Chongqing, the percentage may involve millions of people. Many instances could have influenced the low percentage. There are profit-seeking advertisements in Chongqing TV, unpopular programs, and low audience rating. Mass media campaigns could directly and indirectly produce positive or negative changes in health-related behaviors across large populations [27]. Therefore, the collective experience in campaign research and evaluation showed that health education on chronic disease prevention could be transmitted by a variety of media, especially by the TV channels with high audience rating and popular media China Central Television (CCTV), Internet and mobile phone) to expand public acceptance of the advertisement. Program planners need to carefully examine the relative cost effectiveness of mass media channels they use to promote chronic disease prevention and control and to identify critical points when shifting limited media resources from television to other media.

The aim of mass media campaigns is to encourage and sustain positive behavior change. Public service advertising, with its specific and vivid form, contains a profound moral content as well as the advantages of living close to the audience. Thus, this form of advertising can be easily recognized by people and complete its role in cultural integration [41]. However, there are so many profit-seeking and unhealthy advertisements (e.g., tobacco, alcohol, junk food, sugar-sweetened beverages), which have a negative influence on health. For example, TV television food and sugar-sweetened beverages advertising has influenced children’s preferences and purchasing behavior, which is harmful to the health of children [42,43,44,45]. Alcohol advertising leads to heavy drinking and drinking in dangerous situations [46,47]. Many countries have imposed restrictions on unhealthy advertising, which is needed for China [48,49,50,51]. If the adverting of unhealthy products can be restricted, that may improve the effectiveness of the public service advertising.

The study shows that advertising can prompt participants to consider for chronic disease prevention and control, but cannot show actual behavior change. Most respondents reported the advertising was related to their life, provide new information for them, and make them stop and think. The advertisement was likely to improve respondents to attempt persuading others to change bad lifestyle and remind others to prevent the chronic disease. Although this advertisement did not show differences on related knowledge among we analyzed the study results based on attitude behavior change of the respondents, more than 85% respondents reported that the advertising made them more likely to change their unhealthy lifestyle. This study may provide reference for future chronic disease prevention and control. However, the biggest challenge of health education is to convert knowledge into sustained action. To have a family member make one critical comment about one’s bad lifestyle will not necessarily translate into an individual changing to a healthy behavior path, especially when addiction is involved (e.g., cigarettes, alcohol and other drugs). Further researches combining the sustained intervention and support through clinical and community health programs media campaigns are needed to support public health [52].

The effectiveness of chronic disease prevention and control advertisements among urban population could be influenced by numerous factors in China. For logistic regression analysis, significant differences are explored in occupation and education level. However, overall results indicated few differences in responses to the ad by age, gender, and health condition. As for the daily media exposure frequency of the participants, the television and social network site are popular, this is may also influence effectiveness of the advertising. Further study need to increase the application scope of public service advertisements. At the same time, designers of chronic disease advertising must fully consider the factors that influence the advertisement effect when planning for future advertisements.

### Limitations

This study has certain limitations. First, the cross-sectional survey data reduced the ability to make direct causal inferences, to explore whether unmeasured factors may better explain the observed relationships we observed, and to determine the direction of causality. Second, the questions of the questionnaire are close-ended which cannot measure of what the respondent actual knows and thinks, without being prompted. But the close-ended questions are easier to answer, save time, and less educated respondents complete, and respondents are more receptive to this approach, resulting in higher response rates. Further studies could combine the close-ended and open-ended questions to get more information. Third, due to limitation of design, we only evaluated the awareness and attitude of the advertising, and did not ask the participants who have not seen the advertising to answer the same question with those who have seen the advertising on attitude of chronic disease prevention and control, that cannot be compared in the two groups. And we did not set the question of the actual behavior change in the questionnaire, like eating behavior and smoking or drinking behavior. However, if the participants are willing to discuss this knowledge with others, or to persuade others pour attention into the harm of chronic disease, which may prove that they are willing to change their behavior, and even they have changed their behavior. Future research needs to improve the design, provide more detailed actual behavior to compare the difference of the two groups. Fourth, this study is a street intercept survey, can be called “convenience sample”, which is not representative and generalizable to the entire population—especially home-bound people, elderly, illiterate, and rural. Fifth, there was no baseline data before the intervention, which did not allow for a before-and-after comparison and analysis. Respondents who saw the advertising before were not asked the questions about their knowledge and attitude of chronic disease. Last, many other effects could not have been found in the present research, and such as the time spent on watching the advertisement, level of comprehension, and the form of advertising, which were not taken into account in this study. Further researches and analysis of these factors will help to understand the effect of advertising. And continual monitoring is needed for the effect of chronic disease prevention and control advertising.

## 5. Conclusions

As the first to assess the premier chronic disease prevention and control television campaign in Chongqing, China, the conclusions may be limited to referring to the effectiveness of this advertising in particular. There was minimal change of awareness of the participants who saw the advertisement. This study did not show significant differences on chronic disease related knowledge between the participants who have seen or have not seen the advertisement. After watching the public service advertisement, more than 60% stated that it may help them change future behavior and help them transfer that knowledge to others. Occupation, educational level influenced the effect of the transfer knowledge behavior changes. Future measures are needed to increase the effect of common mass media on the publicity of chronic disease knowledge.

## Figures and Tables

**Table 1:** Demographic characteristics of the participants, Chongqing, China (*n* = 985).

Variables	Seen the Advertisement Entitled “Being Healthy, Being Away from Chronic Diseases”				
	YES	(%)	NO	(%)	*p*-Value
Total	261	26.5	724	73.5	
Age (years)					0.716
24–44	153	58.6	415	57.3	
45–60	108	41.4	309	42.7	
Gender					0.476
Male	131	50.2	382	52.8	
Female	130	49.8	342	47.2	
Residence time					0.789
6–12 months	22	8.4	65	9.0	
More than 12 months	239	91.6	659	91.0	
Having chronic disease					0.076
Yes	40	15.3	74	10.2	
No	184	70.5	531	73.3	
Do not know	37	14.2	119	16.5	
Occupation					0.221
Office staff	119	45.6	287	39.7	
Students	10	3.8	35	4.8	
Farmer	16	6.1	47	6.5	
Laborer	49	18.8	119	16.4	
Professional job	67	25.7	236	32.6	
Education level					0.213
Basic education	81	31.0	274	37.9	
Secondary education	122	46.8	292	83.9	
Higher education	58	22.2	158	21.8	

Occupation was categorized as students, famers, labors, office staff (including government employee and private employee) and professional job (including teachers and medical staff). Education level was categorized as basic education including primary school, junior middle school; secondary education including senior high school, vocational/technical, secondary school, and junior college; and higher education including senior college and university. Chi-square test was used. Statistically significant *p* < 0.05.

**Table 2:** Score of chronic disease knowledge.

Score	Seen the Advertisement Entitled “Being Healthy, Being Away from Chronic Diseases”				
	YES (%)		NO (%)		*p*-Value
					0.210
Low	23	8.8	84	11.6	
Good	52	19.9	166	22.9	
Excellent	186	71.3	474	65.5	
Mean score	18.02 ± 3.12 (SD)		17.60 ± 3.26 (SD)		0.075

The total score of knowledge was classified into three categories on the basis of the quintile scores and coded as “low”, “good”, and “excellent” for ≤60% (<14), 61% to 80% (14–18), and >80% (>18), respectively. SD: standard deviation. Analysis of *t*-test was used. Statistically significant *p* < 0.05.

**Table 3:** People’s awareness of the types of chronic disease and the healthy lifestyle for chronic disease prevention.

	Seen the Advertisement Entitled “Being Healthy, Being Away from Chronic Diseases”						*p*-Value
	Total	(%)	YES	(%)	NO	(%)	
**Types of chronic disease**							
Cardiovascular disease	703	71.4	198	75.9	505	69.8	0.061
Cancer	575	58.4	166	63.6	409	56.5	0.046 *
Diabetes	788	80.0	215	82.4	573	79.1	0.263
Chronic respiratory disease	825	83.8	212	81.2	613	84.7	0.196
**Healthy lifestyle for chronic disease prevention**							
Quit smoking-limited wine	911	92.5	246	94.3	665	91.9	0.207
Balanced diet	908	92.2	243	93.1	665	91.9	0.518
Regular schedule	915	92.9	240	92.0	675	93.2	0.491
Regular exercise	926	94.0	243	93.1	683	94.3	0.472
Healthy state of mind	850	86.3	231	88.5	619	85.5	0.226
Healthy eating	348	35.3	123	47.1	225	31.1	<0.001 *

Chi-square test was used. * Statistically significant (*p* < 0.05).

**Table 4:** Personal subjective assessment of effects of the public service advertising (*N* = 261).

Item	Response	
	*n*	(%)
Effect of the Advertisement on Attitude of Respondents		
The advertising was related to my life	181	69.0
The advertising made me stop and think my life	190	72.8 ^Δ^
The advertising provided new information to me	193	74.0 ^Δ^
I discussed the advertising with others	165	63.2
I noticed the risk factors of chronic diseases	204	78.2 ^Δ^
The advertising made me remind others to prevent chronic diseases	202	77.4 ^Δ^
The advertising made me persuade others to change their unhealthy lifestyle	204	78.2 ^Δ^
The advertising increased the possibility of changing my own unhealthy lifestyle	221	84.7 ^Δ^

^Δ^ More than 70% of the respondents chose the option.

**Table 5:** Logistic regression analysis of the public service advertisement toward the advertisement effects (*N* = 261).

Item	Discussed with Others about this Advertising		The Ad Made Me Remind Others to Prevent Chronic Diseases		The Ad Made Me Persuade Others to Change Their Unhealthy Style		The Ad Made Me More Likely to Change to a Healthy Lifestyle	
	OR (95% CI) ^a^	*p*-Value	OR (95% CI) ^a^	*p*-Value	OR (95% CI) ^a^	*p*-Value	OR (95% CI) ^a^	*p*-Value
Age (years)								
24–44 ^Δ^	1		1				1	
45–60	0.909 (0.482–1.713)	0.767	0.565 (0.263–1.216)	0.144	0.827 (0.392–1.745)	0.618	0.815 (0.339–1.957)	0.647
Gender								
Male ^Δ^	1		1		1		1	
Female	1.173 (0.682–2.018)	0.564	0.603 (0.31–1.176)	0.138	0.821 (0.434–1.554)	0.544	1.394 (0.628–3.095)	0.414
Residence time								
6–12 months ^Δ^	1		1		1		1	
More than 12 months	0.646 (0.241–1.729)	0.384	0.739 (0.218–2.51)	0.628	1.070 (0.356–3.213)	0.905	3.047 (0.980–9.478)	0.054
Having chronic disease								
Yes ^Δ^	1	0.917	1	0.555	1	0.446	1	0.066
No	1.240 (0.449–3.422)	0.678	1.618 (0.438–5.977)	0.471	2.180 (0.621–7.651)	0.224	0.121 (0.011–1.269)	0.078
Don’t know	1.115 (0.505–2.462)	0.787	1.823 (0.613–5.419)	0.280	1.832 (0.644–5.211)	0.256	1.402 (0.410–4.798)	0.590
Occupation								
Students ^Δ^	1	0.096	1	0.637	1	0.760	1	0.115
Office staff	1.241 (0.612–2.515)	0.550	1.07 (1.464–2.473)	0.873	1.651 (0.716–3.807)	0.239	0.293 (0.105–0.822)	0.020 *
Farmer	0.323 (0.056–1.858)	0.206	0.186 (0.019–1.853)	0.152	0.893 (0.145–5.479)	0.902	0.078 (0.007–0.884)	0.039 *
Laborer	1.147 (0.310–4.247)	0.837	1.236 (0.315–4.846)	0.761	1.126 (0.260–4.871)	0.874	0.000	0.998
Professional job	2.412 (1.090–5.338)	0.030 *	1.026 (0.389–2.702)	0.959	1.185 (0.445–3.154)	0.734	0.817 (0.290–2.298)	0.701
Education level								
Basic education ^Δ^	1	0.020 *		0.000 **	1	0.027 *	1	0.002 *
Secondary education	0.282 (0.112–0.709)	0.007 *	0.23 (0.081–0.652)	0.006 *	0.544 (0.198–1.498)	0.239	0.291 (0.085–0.997)	0.049 *
Higher education	0.431 (0.211–0.879)	0.021 *	0.132 (0.056–0.312)	0.000 *	0.340 (0.152–0.758)	0.008 *	0.145 (0.050–0.416)	0.000 *

* Statistically significant (*p* < 0.05). ^a^ Logistic regression model was adjusted for gender, age, education level. ^Δ^ Reference group. Education level was categorized as basic education including primary school, junior middle school; secondary education including senior high school, vocational/technical, secondary school, and junior college; and higher education including senior college and university. Occupation was categorized as students, famers, labors, office staff (including government employee and private employee) and professional job (including teachers and medical staff). OR: odds ratio; CI: confidence interval.

**Table 6:** Daily Contact of mass media of all participants (*N* = 985).

Variable	Seen the Advertisement Entitled “Being Healthy, Being Away from Chronic Diseases”			*p*-Value
	Total (%)	YES (%)	NO (%)	
Television	549 (55.7)	156 (59.8)	393 (54.3)	0.128
Radio	111 (11.3)	35 (13.4)	76 (10.5)	0.210
Newspapers	250 (25.4)	82 (31.4)	168 (23.2)	0.010 *
Magazine	107 (10.9)	42 (16.1)	65 (9.0)	0.002 *
Website	419 (42.5)	113 (43.3)	306 (42.3)	0.771
Social Network Site	426 (43.2)	110 (42.1)	316 (43.6)	0.716

* Statistically significant (*p* < 0.05).
